# Analysis of definitions of general practice, family medicine, and primary health care: a terminological analysis

**DOI:** 10.3399/bjgpopen17X101049

**Published:** 2017-10-04

**Authors:** Marc Jamoulle, Melissa Resnick, Robert Vander Stichele, Ashwin Ittoo, Elena Cardillo, Marc Vanmeerbeek

**Affiliations:** 1 GP, Researcher, and PhD applicant, Department of General Practice, University of Liège, Liège, Belgium; 2 Medical Librarian and PhD applicant, Health Science Center, University of Texas at Houston, Houston, TX, US; 3 GP and Professor of Pharmacology, Heymans Institute of Pharmacology, University of Ghent, Ghent, Belgium; 4 Associate Professor in Health Information Systems, HEC Management School, University of Liège, Liège, Belgium; 5 Senior Researcher in Computational Linguistics, Institute of Informatics and Telematics, National Research Council, Rende, Italy; 6 GP and Professor of General Practice, Department of General Practice, University of Liège, Liège, Belgium

**Keywords:** primary health care, general practice, terminology as topic, qualitative research

## Abstract

**Background:**

There are numerous definitions of general practice/family medicine (GP/FM) and primary health care (PHC), but the distinction between the two concepts is unclear.

**Aim:**

To conduct a terminological analysis of a set of definitions of GP/FM and of PHC, to clarify the commonalities and differences between these two concepts.

**Design:**

Sets of 20 definitions were collected in two 'bags of words' (one for GP/FM and one for PHC terms). A terminological analysis of these two collections was performed to prioritise the terms and analyse their universe of discourse.

**Method:**

The two collections were extracted with VocabGrabber, configured in two 'term clouds' using Wordle, and further explored for similarities using Tropes. The main terms were analysed using the Aristotelian approach to the categorisation of things.

**Results:**

Although continuity of care (characterised by a person-centred approach and shared decision making) is common to both sets, the two sets of definitions differ greatly in content. The main terms specific to GP/FM (*community, medicine, responsibility, individual, problem,* and *needs*) are different from those specific to PHC (*home, team, promotion, collaborator, engagement, neighbourhood,* and *medical* centre).

**Conclusion:**

Terminological analysis of the definitions for GP/FM and PHC shows two overlapping but distinct entities, necessitating a different taxonomic approach and different bibliographic search strategies.

## How this fits in

There are numerous definitions of GP/FM and PHC. The governance of these concepts is related to their use in two distinct organisations: the World Organization of National Colleges, Academies and Academic Associations of General Practitioners/Family Physicians (WONCA) and the World Health Organization (WHO). In GP/FM textbooks and bibliographic retrieval systems, there is often confusion between these concepts. A clear understanding of the similarities and differences between the two concepts is needed for the organisation of medical training, for the development of the profession and of health policy, and for optimal information storage and retrieval in this scientific discipline.

## Introduction

General practice designates a branch of medicine characterised by its broad scope. The term general, also extended to generalism,^1^ encompasses the comprehensive range of transactions performed, and thus the scope and nature of the work of the practitioner.

Family medicine emphasises the relationship with the patient and seeing the person as a whole, in the context of their family (next of kin or relevant others) and their wider community. The WONCA dictionary states: ‘Many medical practitioners in the primary health care prefer the terms family physician and family medicine in order to emphasise the recognition of their branch of medical practice as a specialty in its own right.’^2^ In other countries, other terms are used such as general practitioner (UK), 'hausart' (Germany), 'huisarts' (Netherlands), 'médecin généraliste' and 'médecin de famille' (France), and family physician (US). WONCA has always used the pair of terms GP/FM in order to present and discuss the situation, taking into account the members of this professional organisation. Hence, GP/FM is a people-oriented profession aiming at the management of an extended and general set of human health problems.^3^ Core values of GP/FM have been extensively discussed. Patient-centredness, as well as the biopsychosocial model, are now definitely considered as undisputable attributes of a profession directed towards building personal relationships during the patient’s lifetime.^4^

The concept of primary health care (PHC), endorsed by the WHO in 1978 at Alma-Ata, is an organisational concept.^5^ It addresses the place, management, and workload of the first (primary) level of health care, as well as its inclusion in the network of care facilities. ‘*S*trong primary health care is the foundation of healthy communities’ remains a WHO motto.

The aim of this study was to conduct a terminological analysis of a set of definitions of GP/FM and of PHC, in order to clarify the commonalities and differences between these two concepts.

## Method

To construct a set of relevant definitions for each of the two concepts (GP/FM and PHC), a search of PubMed, Google Scholar, Global Index Medicus, the WHO bibliographic database,^6^ and books related to the discipline was made. For GP/FM, the following keywords were used: *family practice; general practice; general practitioners; physicians, family; physicians,* and *primary care*. For PHC we used: *primary health care; community health centres; community health services; rural health services; *and *home care services.*

Definitions that were repetitive or yielded no further information were disregarded. We aimed for geographical and cultural spread, stopping after 10 definitions for each concept, because new definitions did not provide any additional significant information.

Furthermore, a terminological analysis of these two sets of 10 definitions for GP/FM and PHC was performed to prioritise the terms used in each of the two sets. To this end, we first targeted the key vocabulary in the definitions by using VocabGrabber, a text analysing tool, which ranks the relevance of all of the words appearing in a source text by comparing the frequency of their use in the presented text to their overall frequency of use in written English (https://www.visualthesaurus.com/vocabgrabber). In this system, the more frequent words can be displayed in a tabular list with the numerical frequency and relevance of each word shown, or in a semantic map with a view of the relationships between words and meanings. The relative relevance of terms can be displayed in a 'tag cloud' through the use of a specific 'word cloud' generator such as Wordle (http://www.wordle.net). Here words that appear more frequently in the source text are given greater prominence in the cloud (they appear in a larger font).

In addition, we used Tropes, a natural language processing software program designed for semantic classification, keyword extraction, and linguistic and qualitative analysis (http://tropes.fr/).

Finally, the prioritised terms within each set of definitions and their semantic relationships were then used to perform a comparative analysis of the two concepts (GM/FM and PHC). To clarify what links and what separates the two concepts, we used the classical category theory approach of Greek philosopher Aristotle (4th century BCE), in which the meaning of a term is explored by asking 10 fundamental questions about the universal categories of things:

1. essence;
2. quantity;
3. quality; 
4. relation;
5. place;
6. time;
7. posture;
8. state;
9. action; and 
10. passion.

This approach is still used, for example, in the development of taxonomies and ontologies to identify relevant concepts of a domain of application and to categorise these concepts.^7,8^

## Results

Twenty definitions (10 relating to GP/FM and 10 relating to PHC) were selected from the results of a larger, exhaustive search. These definitions were in English, Spanish, Portuguese, and French, spanning Europe, the US, Canada, South America, Australia, and India. The dates of the 10 GP/FM definitions ranged from 1974 to 2016, while those of the 10 PHC definitions ranged from 1996 to 2016 (Box 1).

**Box 1.** Sources of the two sets of 10 definitions of GP/FM and PHC.

General Practice/Family Medicine definitions	Primary Health Care definitions
Leeuwenhorst definition (1974)^9^	Institute of Medicine (1996)^10^
AAFP primary care physician definition (US) (1977)^11^	PAHO primary health care statement (Pan America) (2007)^12^
AAFP family medicine definition (US) (1984)^13^	EU expert panel definition of primary care (2014)^14^
Olesen’s proposal for a new definition of general practice (2000)^15^	Brazil: organisation of primary health care (2013)^16^
WONCA dictionary (2003)^2^	WHO glossary (2016)^17^
WONCA Europe / EURACT definition (2011)^18^	PHCRIS (Australia) (2015)^19^
CIMF Carta de Quito definition (Latin America) (2014)^20^	FMMCSF (Belgium) (2016)^21^
The Role Definition Group definition (US) (2014)^22^	AHRQ Primary Care Medical Home model (US) (2016)^23^
NBE definition (India) (2015)^24^	FFMPS (France) (2016)^25^
AAFP Primary Care Physician (2016)^11^	AAFP Primary Care (US) (2016)^11^

AAFP = American Academy of Family Physicians. AHRQ = Agency for Healthcare Research and Quality. CIMF = Confederación Iberoamericana de Medicina Familiar / Iberoamerican Confederation of Family Medicine. EU = European Union. FMMCSF = Fédération des Maisons Médicales et des Collectifs de Santé Francophone / Federation of Medical Homes and French-speaking Health Centres. FFMPS = Fédération Française des Maisons et Pôles de Santé */* French Federation of Housing and Health Centers. NBE = National Board of Examination. PAHO = Pan American Health Organisation. PHCRIS = Primary Health Care Research and Information Service. WHO = World Health Organization. WONCA = World Organization of National Colleges, Academies and Academic Associations of General Practitioners/Family Physicians.

**Figure 1.**
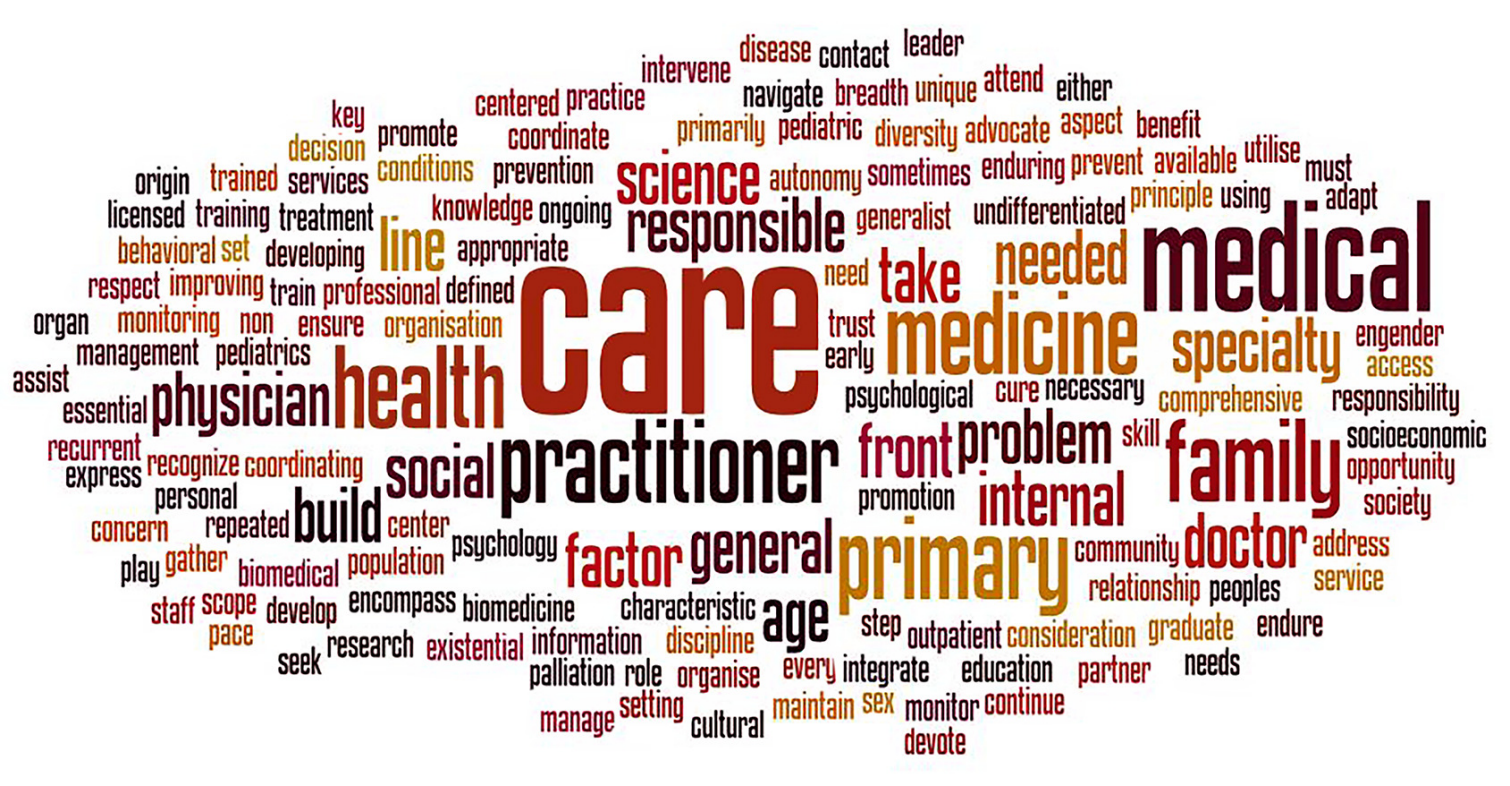
Tag cloud for General Practice/Family Medicine.

**Figure 2.**
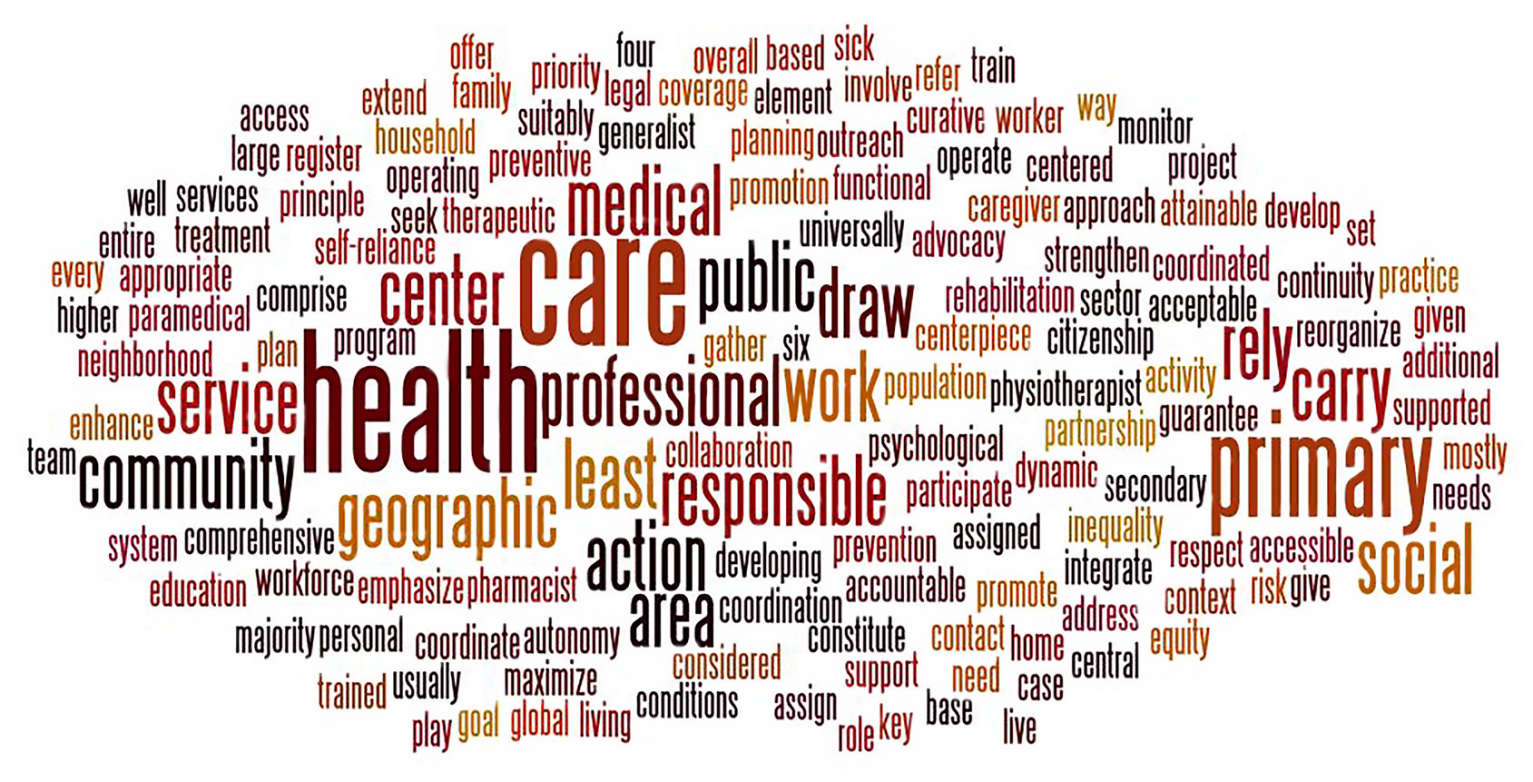
Tag cloud for Primary Health Care.

Using the above-mentioned VocabGrabber tool, 319 words were taken from the GP/FM set of definitions, and 262 words from the PHC set, and displayed in two tag clouds generated using Wordle Figure 1 and Figure 2.

Words or compound words mentioned at least three times in both sets of definitions, or appearing in only one set, analysed using Tropes software, are outlined below (Box 2).

**Box 2.** Terms that unite and separate the two concepts: GP/FM and PHC.

**What unites**	Listed at least three times in both sets	Care, health, patient, service, family, community, health care, system, prevention, doctor, population, needs, provision, junction.
**What separates**	Listed at least three times only in GP/FM set	Medicine, responsibility, individual, general practitioner, sex, illness, disease, problem, peculiarity, specialist, factor, management, science, basis, age, resource, point.
	Listed at least three times only in the PHC set	Home, team, promotion, person, part, activity, health professional, righteousness, nurse, majority, action, professional, partnership, access, level, improvement, time, insurance, collaborator, engagement, neighbourhood, medical centre.

Finally, the main terms were analysed, using the 10 seminal Aristotelian categories of things,^26^ and integrated to a statement in response to the philosophical questions, relevant for each category, for each of the two sets (Box 3).

**Box 3.** Analysis of the distribution of the main terms of the 10 definitions of GP/FM and PHC according to Aristotle's categories of things.

Aristotelian categories Greek and Latin translations	Question	General Practice/Family Medicine words	Primary Health Care words
Essence *οὐαία* (*ousia)* *Quod est? Essentia*	What is it? Essence or substance?	Licensed medical graduate who provides care, specialty charterised by breadth, primary care services, take care, promotion of health, prevention of disease, early diagnosis, initial decision, provision of clinical care, rehabilitation, palliative care, education, research	Clinician provides healthcare services, care, health, prevention, promotion, first-contact primary care, intersectoral action, health promotion, illness prevention, treatment and care of the sick, community development, rehabilitation
Quantity *Πoσόν (poson)* *Quantum, Quatitas*	How much, how many, how tall?	General, every, both sexes, all age, irrespective of age, each organ system, every disease entity, repeated contacts, entire family	Large majority, any, set, variety, every family, entire population
Quality *Ποιόν (poion)* *Quale* *Qualitas*	How is it? What kind or quality?	Personal, access, available, comprehensive, effective, necessary, personal, respecting autonomy, safety, satisfaction, sustainability	Universal coverage, comprehensive, integrated, person-centred, accessible, socially appropriate, critical, effective, scientifically sound, include, partner, professional, specifically, undiagnosed, undifferentiated, whole-person care
Relation *πρός τι* *(pros ti)* *Relativum*	What is it related to? Towards something?	In the context of their family, their community, and their culture, family doctor, general practitioner, individual, population, undifferentiated patient, cultural diversity	Multiprofessional health teams, partnership, patient, caregivers, population, family, communities, local network
Place *ποῦ (pou)* *Ubi*	Where?	Where necessary, at the point of first contact, entry point, in the front line, consulting room, homes, acute and chronic care settings	Set of functional and structural elements, home, office, setting, coverage area, geographic, territory
Time *Πότε (pote)* *Quando*	When?	First contact, prolonged contact, continuing, repeated, maintaining, always, preventing, chronic, recurrent, terminal	First contact, first level, over time, prevention, primary, continuing, acute, chronic, limited, long term
Posture *κεῖσθαι (keisthai)* *Situ*	From what action does it result?	Autonomy, balance, basis, clinical, contact, cultural, disease, existential, health, illness, needs, self, physical, biomedical, psychological, social and behavioural sciences	Autonomy, behavioural, biological, communication, concern, consultation, contact, disease, health, illness, organ, problem, sign, social, symptom, living conditions, health risks, health status, health inequalities
State *ἔχειν (echein)* *Habitus*	What is it required to have or be?	Socially responsible, reliable, leader, professional, advocate, trust, knowledge, personal balance and value	Equity enhancing, responsible, concern, accountable, cost, role, professional, self-reliance, participation and control, advocacy, social justice, equity, solidarity
Action *ποιεῖν (poiein)* *Agere*	What is it doing? (change), to make or do	Provide, train, integrate, intervene, promote, maintain, prevent, serve, manage, practice, define, optimise, negotiate, coordinate, monitor, devote, gather information, organise, assist	Perform, participate, measure, utilise, monitor, understand, reorganise, maximise, collaborate, assess, inform, integrate, gather, encourage, enable
Passion *Πάσχειν (paschein)* *Pati*	How is it being acted on (be changed)?	Must be trained, developing and maintaining their skills, personal balance and values, discipline, professional role	Accomplish, appropriate, perform, skilled, trained

Both sets share the terms *continuity of care*, *patient centredness*, *community health*, and *shared decision making*. Although *care* is the central issue of the two sets, they differ greatly in content. As indicated in Box 2, the main terms specific to each set differ greatly. GP/FM is determined by such terms as *medicine, responsibility, individual, problem, disease,* and *peculiarity.* PHC is quite service oriented with *home, team, promotion, collaborator, engagement, neighbourhood,* and *medical centre.*

Among the top 10 terms for both GP/FM and PHC concepts, the terms *global health*, *environmental hazard*, *ethics*, economic aspects, and the recent concept of quaternary prevention (danger of overmedicalisation)^27^ are almost absent. None of the definitions specifically addressed medical anthropology. Only in the GP/FM definition from Latin America (‘*Carta de Quito*’ [letter from Quito]),^20^ are the terms *sustainability* and *social responsibility* mentioned.

## Discussion

### Summary

To the best of our knowledge, this is the first terminological analysis of the terms used to depict workforce and structure of primary-level care as found in published definitions of GP/FM and PHC.

Although continuity of care (characterised by a person-centred approach and shared decision making) is core to the two sets, the two sets of definitions differ greatly in content. The main terms obtained from an analysis of 10 definitions of GP/FM pertain to a professional discipline, conducted by *practitioners* who are *responsible physicians* shaped by *science* and who care for *family problems* in the context of a *social* role.

The main terms from the 10 definitions of PHC still speak of *care* and *health* as central elements but, here, it is a *service* to the population made by *unspecified professionals* in a *geographic area*.

### Strengths and limitations

This study provides an innovative method to examine the nature of GP/FM and PHC through a terminological analysis.

The prioritisation of terms based on software tools may be subject to variation over time, as tools evolve. The qualitative interpretation of the terminological findings is a potentially subjective process that needs further validation.

### Comparison with existing literature

As stated by Olesen (2000) and Pereira Gray (2017), many definitions confuse the setting with the role and the person.^15,28^ However, the American Academy of Family Physicians (AAFP) clearly distinguishes between the two concepts, arguing that '... the terms "primary care" and "family medicine" are not interchangeable’.^11^As stated on the website of the WHO Primary Health Care Performance Initiative, PHC is deeply embedded in the following main values: people's first contact, people-centred, comprehensive, continuous, coordinated, accessible (also echoed by a Canadian analysis of 25 attributes of PHC).^29,30^ Worldwide, general practitioners and family physicians, referring to comprehensiveness, personal and patient-centred care and universal accessibility, provide and sometimes organise primary care in PHC settings.^31^ In this terminological analysis, we also found that the two concepts (GP/FM and PHC) are related but distinct.

### Implications for information science and health policy

This terminological analysis of the definitions of GP/FM and PHC may have implications on the construction of field-specific filters for bibliographic searches (for example, a GP/FM filter, a PHC filter). In the filters usually published in the literature, the two concepts tend to be mixed.^32,33^ The present study is part of the development of a taxonomy for the organisational aspects of the activities in GP/FM, as an extension of the International Classification of Primary Care (ICPC-2)^34^ for contextual professional aspects.^35^

This study may facilitate a dialogue between the two organisations, which have pioneered these two concepts and are still governing them, that is WONCA for GP/FM, and WHO for PHC. These organisations could come to a better understanding of the commonalities and complementarities of their endeavors, to foster mutual collaboration.^36–38^ In addition, it was observed that in both sets of definitions important aspects are missing. Environmental issues are very poorly addressed as are ethical challenges. Those are numerous and are a core task for general practitioners (for example, ethics of information and ethics of prevention).^27^ There is also a need to adapt the definitions to take into account 21st century insights and developments in information and communication technology. Both organisations should collaborate to produce updated, profound and distinct definitions for both GP/FM and PHC.
